# The evolution of the concepts of seizures and epilepsy: What’s in a name?

**DOI:** 10.1002/epi4.12375

**Published:** 2020-01-10

**Authors:** Puja Patel, Solomon L. Moshé

**Affiliations:** ^1^ Isabelle Rapin Division of Child Neurology of the Saul R Korey Department of Neurology Department of Pediatrics and Comprehensive Einstein/Montefiore Epilepsy Center Albert Einstein College of Medicine and Montefiore Medical Center Bronx NY USA; ^2^ Dominic P. Purpura Department of Neuroscience and Laboratory of Developmental Epilepsy Albert Einstein College of Medicine Bronx NY USA

**Keywords:** classification of seizures/epilepsy, hallmarks in history of seizures/epilepsy, seizures/epilepsy in antiquity

## Abstract

This review aims to highlight the historical hallmarks in the development of the concepts of seizures and epilepsy. It begins with a discussion of seizure semiology and terminology, followed by the pathophysiology of seizures. We then discuss the definition of epilepsy, its etiologies, and ultimately classification schemes. Each section starts with our current views and subsequently transports the reader back in time to understand how these views evolved and came to be what they are today. People living as early as in the prehistoric times may have been aware of the existence of seizures, and descriptions and terminology have been provided as early as 2500 BC. While names have been revised and updated through time, the meanings are seemingly unchanged. However, it is clearly evident that we have come a long way in understanding the pathophysiology and etiology of seizures and epilepsy, thus leading to our current classification schemes. No classification scheme will be perfect yet, until our understanding is advanced enough to create one based predominantly on scientific grounds. The goal is that it is relevant to clinical practice, leading to a more precise diagnosis to guide targeted treatments.

**Key Point**

- The objective of this review is to provide a historical trajectory in the evolution of the concepts of seizure and epilepsy
- Seizure descriptions and terminology are documented as early as 2500 BC; names have been revised and updated through time but meanings have not changed
- Our understanding of the pathophysiology and etiology of seizures and epilepsy has remarkably advanced, leading to the development of classification schemes
- The goal of seizure and epilepsy classifications is that it is relevant to clinical practice and allows us to find a precise diagnosis to guide targeted treatments

## 1 INTRODUCTION

The history of seizures and epilepsy may date back to prehistoric times, perhaps as early as the late Paleolithic period. Beliefs on the causes of seizures coincided with the prevailing concept of religion and medicine of that era, with ideas changing over time from a magical to scientific explanation.^1^ The purpose of this review is to provide the reader with a history on the development of the concepts of seizures and epilepsy. A disease is a pathological condition of a body part, an organ, or a system resulting from various causes, and characterized by an identifiable group of signs and symptoms. To comprehend how a disease came to be, we must begin with a description of the signs and symptoms, followed by names and definitions, which is intimately tied with an understanding of the pathophysiology. What then logically ensues is an understanding of causes or etiology. Ultimately, this allows us to create classification schemes. In this review, there are several references describing historical perspectives (including Owsei Temkin's excellent review of the history of epilepsy in Western civilization from ancient times to the beginnings of modern neurology,^2^ among various others), which we will identify with an * to denote “as cited in.”

## 2 TERMINOLOGY AND SEIZURE SEMIOLOGY

Our current description of seizures is guided by the 2017 International League Against Epilepsy (ILAE) revised classification of seizure types.^3^ A seizure is first described by type of onset, which includes focal, generalized, or unknown. A seizure of unknown onset may have defining characteristics; however, due to lack of information, it cannot be confidently classified as focal or generalized. This can be followed by stating the degree of awareness, specifically for a focal seizure, as it is assumed that the large majority of generalized seizures are associated with impaired awareness. Seizures from all three categories may be further classified as motor or nonmotor onset, each with additional descriptors based on the first prominent sign or symptom. Seizures of unknown onset can also be unclassified due to seizure patterns that do not fit into the other categories, or insufficient information for categorization. Focal to bilateral tonic‐clonic is reserved as a separate seizure type. Included in this proposal is a glossary of terms, focused on the aspects of language pertinent to seizures, updated from the 2001 ILAE glossary.^4^

Going back to the beginnings, it seems that the historical descriptions of seizures are seemingly not that different from our descriptions today, and what has transpired through time is assigning terminology. It is important for the reader to note that the terms *epilepsy, epileptic seizure, attack*, or *convulsion* have been used interchangeably throughout the historical literature. This section will focus on seizures as we view them today as a *symptom*, despite the historical literature using at times the term *epilepsy*, which we use today to mean the disease and will be the focus of a later section. The word *seizure* is derived from the Greek meaning “to take hold.” The earliest description of seizures according to a review of the historical literature is found in the Sumerian documents dating back to around 2500 BC from Mesopotamia. The text describes a person whose neck is turned, with extremities tense, with eyes open, with frothing at the mouth, and with loss of consciousness. We may call this a focal unaware tonic seizure today, but the people of that time called this *antašubbȗ* (Sumerian term meaning “the falling disease”), related to the hand of Sin and God of the moon.^1^^*,^ 2 ^*^ One of the oldest Babylonian medical texts, *Sakikku* (English translation: “All Diseases”), dating from around 1050 BC, includes the reportedly oldest written account of epilepsy, as it was then perceived and understood. They use terms such as *miqtu* (Akkadian (Babylonian and Assyrian considered as a single culture or language), translating to “the falling disease”), *ṣibtu* (meaning “possession),” and the related verb *ṣabātu*, (meaning both “to seize” and “to possess”). The tablet contains descriptions of what we would call today focal onset, tonic, and absence seizures, as well as descriptions of prodromal symptoms, auras, postictal phenomenon, interictal emotional disturbances, and seizure precipitants.^5^^*^

During the Hippocratic and post‐Hippocratic era, a time marking the beginning of scientific views on the origins of seizures and epilepsy, the detailed descriptions of the epileptic attack varied greatly among different authors of the time as per Temkin. Features common to all forms of the epileptic attack included a fall to the ground, unconsciousness, insensibility to pain, and no recollection of the attack upon regaining consciousness. Further symptoms depended on the type of fit.^2^^*^ Aretaeus of Cappadocia (circa 1st/2nd century AD) likened a bilateral tonic‐clonic seizure to the movements of a slaughtered animal (postdecapitation seizures have been discussed in the animal models of epilepsy^6^) and the foam at the mouth to that of the sea. After the fall to the ground, he distinguished three main periods of an epileptic crisis. These included the following: the manifestation characterized by insensibility and convulsions; the abatement characterized by discharge of urine, excrements, and semen, and frothing at the mouth; and the cessation characterized by signs of physical and psychic discomfort. He was reported to be the first to describe an aura as luminous circles of diverse color, ears ringing, smell of bad odors, tremors, and sensations in the hands or feet that may occur before the seizures.^7^^*,^ 8 ^*^ If no convulsions are present, the patient lies pale and motionless in a deep sleep, likened to apoplexy. The description of this type has been combined from the Anonymous Parisinus (1st century AD) and Caelius Aurelianus (5th century AD).^2^^*^ Galen's (129‐201 AD) observations led him to categorize epilepsy as originating from the brain (“idiopathic”) versus other body parts (“sympathetic”) with subsequent involvement of the brain, based on the behavioral manifestations of a seizure. His explanation of sympathetic epilepsy came from his observations from patients who reported symptoms such as palpitations, abdominal sensations, numbness or tingling prior to or as the initial symptoms of their epileptic attack.^2^^*,^ 9 ^*^ It has been said that he introduced the concept of an aura (Greek, meaning “breeze”) into medical terminology, when he referred to a patient who described the initial symptoms of his attack as a sensation of a cold breeze moving upward from his legs to his head.^10^^*^

During the Middle Ages (476‐1492), the scientific views on epilepsy experienced a setback, and the idea of demonic possession again dominated the thinking. Literature of this time uses terms including “falling evil,” “demon,” and “lunacy”; the latter reflected the effect of the moon (luna in Latin) and evil spirits related to it. The description of seizures during this time was vague, causing confusion between epilepsy and mental disorders.^2^^*,^ 11 ^*^ However during this time in the Middle East, Ibn‐e‐Sina, a Persian physician (who is commonly known as Avicenna (980‐1037 AD) in the west‐centric world) in the Golden Age of Islam, made contributions to the field of epileptology in his masterpiece book *Al‐Qanun fi al‐Tibb* (Canon of Medicine). He believed that the clinical manifestation of a seizure may be associated with its origin (brain, stomach, spleen, the *“Maraqq”* defined as a membranous structure in the abdomen, and the whole body) or related to a specific humor (explained below). He described seizures presenting with temperament changes (“to a state similar to melancholy” and distraction accompanied by violent reactions) and psychoneurological malfunctions prior to seizures such as strong anxiety and excitement. He marked the cases similar to the falling sickness where there were no convulsions, referring to what we would think of today as a nonconvulsive seizure. He also alluded to the paroxysmal character of seizures.^12^^*^

The Renaissance (1300‐1600) opened a period of debate, and new observations broadened clinical knowledge. The observation that epilepsy (seizures) was a manifestation of some newly discovered diseases, such as syphilis, scurvy, smallpox, and measles, led to the concept of *symptomatic* epilepsy, where “symptom” meant complication rather than a sign.^2^^*,^ 13 ^*^ The relationship between vertigo and epilepsy was also questioned; sometimes physicians were not able to distinguish between the two. There were also difficulties in differentiating between hysterical and epileptic attacks, especially in cases where epilepsy was supposed to originate from the uterus. It was no longer thought to be necessary for the whole body to convulse or even to fall to the ground for a person to experience a seizure. Convulsions may be pronounced on one half the body only, or slight movements of the extremities, a short state of confusion, laughter, or a change in expression could be the sole manifestations.^2^^*,^ 9 ^*^ It is perhaps here that the idea of focality was becoming recognized in the medical literature. This was furthered during the Enlightenment period (1715‐1789) when William Cullen (1710‐1790) accurately described that convulsions may only affect certain parts of the body and not involve a loss of consciousness.^14^ Gerard Van Swieten (1700‐1772) described at length the clinical characteristics of different types of seizures and tried to explain symptoms such as screams and salivation.^15^ Samuel Auguste Tissot (1728‐1797) apparently wrote the first modern book on epilepsy, introducing terms such as *grands accés* and *petits accés* (English translation of accés: bout, outburst, outlet, upsurge, eruption). He seemingly provided the first detailed description of absence seizures (*petits accés*) that are commonly seen in childhood absence epilepsy.^16^

During the 19th century, with the changing attitudes about epilepsy, patients suffering from the disease were segregated from criminals and the insane in asylums, leading to the development of dedicated colonies and hospitals to care for these patients in western Europe and America. This allowed clinicians to closely observe them and introduce a new lexicon to describe seizures. Jean‐Étienne Dominique Esquirol (1772‐1840) stated that attacks alternate in intensity and there are severe (*le grand*) and slight (*le petit mal*) attacks, though his definition of the terms was vague.^2^^*,^ 17 Louis‐Florentin Calmeil (1798‐1895) reportedly familiarized the term “absence,” which he characterized by a passing mental confusion without any definite physical symptoms, and differentiated this from “petit mal,” which remained the term to include the various attacks that did not have the character of “grand mal.” He was also said to be the first to differentiate between severe fits and *état de mal*, or seizures occurring uninterruptedly (now known as status epilepticus). The definition of an “aura” that had been adopted to mean an ascending cold breeze by Galen was doubted by some physicians of this time and broadened to include all possible warning signs.^2^^*^ James Cowles Prichard (1786‐1848) purportedly coined the term “partial epilepsy”; however, it is debatable as to whether he meant to use this term to describe an incomplete attack or one that is localized to part of the body, the latter with an intention similar to the modern one and which some believe should be credited to Cullen.^18^^*,^ 19 William James West (1794‐1848) described clearly the symptoms of what we now know as infantile spasms in his own son, then called “salaam convulsions” by Sir Charles Clarke whom West brought his son to for a consultation.^20^

It was during the time of John Hughlings Jackson (1835‐1911) that the field of neurology and epileptology was beginning to establish itself. Jackson used the observations and ideas of several of his forerunners and combined them with his own to create his mark in history. Théodore Herpin's (1799‐1865) statements on incomplete attacks in his monograph were later quoted and supported by Jackson.^21^^*^ Louis Francois Bravais (1801‐1843) aimed at establishing a new type of epilepsy, which he tried to distinguish from generalized epilepsy, and called “hemiplegic epilepsy,” where only one side of the body was attacked by convulsions often followed by more or less severe paralysis of the affected side. He did not go beyond descriptions however, and it was not until Jackson's insights that this work was said to have become more noticed.^22^^*^ Robert Todd (1809‐60) used the term “epileptic hemiplegia” for cases similar to those described by Prichard and Bravais, now called Todd's paralysis.^23^ Jackson's work, like that of his forerunners, related to unilateral seizures or epilepsy. He used different terms based on where in the brain he thought the seizure originated. The brain was divided into three levels: He termed lowest level fits “pontobulbar fits” (or brainstem seizures) and represented rough and simple movements; middle level fits from the sensorimotor cortex “epileptiform seizures” and represented complex movements of all parts of the body; and highest level fits from the frontal lobes or the organ of the mind “epileptic seizures.”^24^ He discussed how seizures marched from one part of the body to other parts (Jacksonian epilepsy), what we may now call focal aware motor seizures. Jackson also elaborated on seizures starting with a dreamlike state and the “uncinate group of fits,”^10^^*^ today called a focal aware/impaired awareness cognitive or sensory seizure.

With the advent of EEG in the late 19th and early 20th century, the understanding of epilepsy profoundly increased, which was accompanied by more accurate clinical descriptions. The dichotomy between “focal” and “generalized” was further supported by the finding of two fundamentally different types of epileptiform EEG patterns observed.^21^ Frederic Andrews Gibbs (1903‐1992) and his colleague Erna Leonhardt‐Gibbs (1904‐1987), together with William Lennox (1884‐1960), distinguished different EEG patterns for the three major types of clinical seizures including petit mal (what we call today absence seizure), grand mal (what we call today generalized tonic‐clonic), and psychomotor seizures (or focal seizures arising from the temporal lobe; many of these patients had been diagnosed as “hysterical” prior to the introduction of EEG). Furthermore, they discovered that the interictal EEG of many of these patients was often abnormal, allowing the clinician to diagnose a type of seizure or epilepsy syndrome without actually having observed a seizure in that patient.^25^

It became apparent that the classification of seizures up until this time varied considerably and there was a need for a standardized system. In 1964, the International League Against Epilepsy (ILAE) led by Gastaut developed a classification system of seizures. Seizure‐type categories included partial (subdivided into elementary and complex symptomatology, and secondarily generalized), generalized (subdivided into nonconvulsive and convulsive), unilateral or predominantly unilateral in children, erratic in newborn, and unclassified. Gastaut acknowledged the terms partial, focal, and local were used interchangeably; however, he gave the preference to the term partial because he thought it was the oldest and most widely used at that time, and felt to be more correct in the sense that the discharging neuronal population is widely located throughout a region of the brain so cannot be properly described as focal or local.^26^ In an addendum, Gastaut and Broughton stated seizures may also be classified according to frequency (isolated, repeated, prolonged or repetitive).^27^ In 1981, a revised seizure classification was proposed and greatly aided by the advent of video‐EEG recordings, allowing epileptologists around the world to study seizure semiology to develop a common language of terms. The dichotomy of partial versus generalized seizures remained; however distinct from the previous classification, partial seizures were divided into complex and simple depending on whether or not consciousness was impaired.^28^

With the growing understanding of seizures and epilepsy brought about by advances in neuroimaging, molecular and cellular mechanisms, and new therapeutic options necessitated the further refinement of how we classify seizures. In 2001, a Task Force on Classification and Terminology was appointed by the ILAE. They introduced a standard glossary of terminology for ictal semiology^4^ and proposed a diagnostic five‐axis scheme for people with epileptic seizures and epilepsy. As part of this scheme, they suggested ictal semiology using this standardized Glossary of Descriptive Ictal Terminology, and seizure type or types, derived from a list of accepted seizure types, with localization specified when this is appropriate, and in the case of reflex seizures, the specific stimulus. Terms used to previously describe seizures were criticized, including partial (as it implied part of a seizure), simple, and complex, given the lack of public understanding.^29^ Although the ILAE General Assembly approved this new diagnostic scheme, this work did not negate the 1981 classification of seizures, which was used by many. The next major revision in seizure classification was issued in 2010 by Berg et al^30^ Terms that were felt to be misused or misunderstood were replaced. This included the term “focal,” which was used to replace “partial.” The terms “simple” and “complex” were eliminated, and it was recommended to describe focal seizures according to the degree of impairment during the seizure (with or without impairment of consciousness/awareness). Furthermore, the term “secondary generalized seizure” was replaced by “evolving to a bilateral, convulsive seizure.” Other changes included that neonatal seizures were no longer regarded as a separate entity, the subclassification of absence seizures was simplified and altered, and spasms were explicitly acknowledged as a seizure type. This classification and new terminology again met criticism, as some deemed it unnecessary, and others found it may be too complicated to be used in daily clinical practice.^31^, ^32^

We now come back to our current seizure classification scheme, derived for practical clinical use. It is acknowledged by the ILAE that because we continue to lack a fundamental pathophysiologic understanding of differing seizure presentations, grouping of seizure types reflected an operational opinion.^3^ While no classification system at this point will be perfect, the goal is to create a universal language among physicians, patients, and caregivers, to improve communication of symptoms, diagnosis, and management. Descriptions of symptoms is the first step in diagnosing a disease, and in the case of seizures, these descriptions have been around since ancient times. How we call these symptoms is what has evolved and been debated through time, as we gain a better understanding of the disease.

## 3 PATHOPHYSIOLOGY OF SEIZURES

Definitions of symptoms and diseases ideally include assertions about pathophysiology. The ILAE and the International Bureau for Epilepsy (IBE) currently define an epileptic seizure as a transient occurrence of signs and/or symptoms due to abnormal excessive or synchronous neuronal activity in the brain. It is a clinical event with a wide range of possible manifestations.^33^ This definition includes assumptions of both the substrate of a seizure (abnormal excessive or synchronous neuronal activity) and its origin (the brain). The pathophysiology of a seizure may also include theories on propagation patterns.

Our understanding of the pathophysiology of seizures has undergone quite a remarkable evolution through time, with much of the historical literature centered on debates of the origin and substrate of seizures. It was during the time of Hippocrates that a scientific explanation for epilepsy or seizures was first proposed, refuting the then popular magical thinking as the cause. *On the Sacred Disease*, a book in the Hippocratic collection of medical writings dating to around 400 BC was written by an unknown physician and the first attempt to view epilepsy in a scientific and rationale way. It was stated that the seat of the disease lied in the brain, “an organ of senses, motion and intellect.” It was caused by an overflow of phlegm (Table 1) in the brain, which rushes into blood vessels of the body to cause all the symptoms of the attack. Another theory of that time was that of Aristotle (384‐322 BC) who suggested that an excess of black bile produced seizures.^11^^*^ He also compared sleep and epilepsy and believed that food produced vapors, which moved through veins and reached the brain during sleep, causing epilepsy.^34^^*^ Praxagoras (circa 340 BC) proposed that the aggregation of phlegmatic humors in the aorta (which he believed to be the central organ of intelligence and the seat of thought) caused epilepsy, by blocking the passage of “psychic pneuma” (a term to describe an air‐like substance serving as the underlying layer for the mental functions) from the heart, which in turn makes the body shake and convulse.^2^^*^

**Table 1:** The four basic humors

Humor	Element	Secreting organ	Temperament
Blood	Air	Heart	Sanguine—courageous, hopeful, playful, carefree
Phlegm	Water	Brain	Phlegmatic—calm, thoughtful, patient, peaceful
Yellow Bile	Fire	Liver	Choleric—ambitious, leader‐like, restless, easily angered
Black Bile	Earth	Spleen	Melancholic—despondent, quiet, analytical, serious

Note

Hippocrates based medicine on the idea that nature was made of four basic elements, according to the philosopher Empedocles (~493‐433 BC). In the body, they are effective in four body fluids or humors. Health is therefore the harmony of these humors and results in a state of *eukrasia*. An imbalance leads to a state of *dyskrasia* or disease. The ratio of humors in the human body also influences temperament.^72^, ^73^

Physicians and philosophers of the post‐Hippocratic era fostered these scientific views and continued to contribute different theories about epilepsy or seizures. Erasistatos (304‐250 BC), a student of Aristotle, believed epilepsy resulted from an excess of blood in the veins. When pathological conditions prevailed, the amount of blood in the veins might increase and cause a “plethora” (derived from Greek, literally: an excessive amount of something), which in turn would cause various diseases according to its anatomical localization. Galen was also a proponent of the humoral theory and integrated this with the concept of the psychic “pneuma” (derived from Greek, literally: a current of air) to explain his concepts of “idiopathic” and “sympathetic” epilepsy. Pneuma was created in the lungs and acquired its psychic properties in the cerebral ventricles where the “leading faculties” of volition and memory reside, and, through the spinal cord and the nerves, accepts sensations and carries the soul's commands to the voluntary muscles. Galen postulated that the accumulation of viscous humor, either phlegm or black bile, in the ventricles obstructed the flow of psychic pneuma through the ventricles, which produced the loss of awareness and memory seen with a seizure. Additionally, the viscous humor irritated the roots of the nerves, which shake violently to free themselves, and is transmitted to skeletal muscles, thus producing the convulsive movements. Less commonly, however, the original lesion was located elsewhere in the body and the seizure was the result of subsequent involvement of the brain, which he used to explain “sympathetic” epilepsy. He further subdivided this category into epilepsy originating from the cardia (in persons with an abundance of bile and weakness of the stomach, or atony of the cardia) or any other part of the body that resulted from a distal disturbance in the pneuma and propagated to the brain.^2^^*,^ 9 ^*^

During the Renaissance, new scientific theories on the pathophysiology of epilepsy were proposed. Paracelsus (1493‐1541), a Swiss physician, alchemist, and astrologer, took a complex view based on the balance of elements within man, the microcosm, and the outer world (fire, air, water, earth), the macrocosm. He compares a thunderstorm to an epileptic attack. To cure it would entail separating the *corpora*, or the material from which the elements are born.^2^^*,^ 13 ^*^ Charles Le Pois (1563‐1636) refuted the traditional theory of idiopathic and sympathetic epilepsy and proposed that all epilepsies originated in the brain, from a superfluity of serum in the head that flows into the roots of nerves to fill and distend them, causing various motions of the body or affecting the senses.^1^^*,^ 2 ^*,^ 10 He based his argument partly on anatomical findings, which he observed in his postmortem examinations. The lack of ventricular obstruction in most cases suggested that an irritation of the brain or its membranes is what caused seizures.^11^^*^ This irritation theory was further supported with new discoveries in the fields of chemistry and physics in the latter part of the 17th century. Sylvius (1614‐1672) proposed that the irritating material is acid spirits or vapors mingling with animal spirits. Thomas Willis (1621‐1675) came up with the idea that animal spirits released vitriolic chemical particles into the blood, which spill into the brain and spinal cord, irritating the nerves and making the muscles explode.^1^^*^ Giorgio Baglivi (1668‐1707) developed a mechanical theory and wrote about disturbed elastic equilibrium of fibers in the dura mater as the cause of seizures.^10^^*,^ 35 ^*^ Toward the end of the 17th century, Stahl (1660‐1734) reportedly opposed these mechanical theories and viewed the epileptic attack as a reaction of the soul. He distinguished symptomatic convulsions as complications of some disease from epileptic convulsions, which have no relation to other diseases.^2^^*^

In the 19th century, the origin of seizures continued to include both the brain and other body parts, and for those originating in the brain, pathologists debated locations within the brain with close to 2000 anatomical abnormalities observed including the pituitary, medulla oblongata, cerebral hemisphere proper, among many others; however, no uniform results were obtained. Marshall Hall (1790‐1857) proposed the reflex theory, dividing epilepsy into a centric origin in the medulla itself or eccentric where the exciting cause was distant from the nervous centers and forms a reflex arc, leading to a secondary affection of the brain.^2^^*,^ 36 Jean Pierre Flourens (1794‐1867) refuted that the cerebral hemispheres and cerebellum were irritable, but instead postulated irritability pertained to the spinal cord, its continuation (medulla oblongata), and end (quadrigeminal plate), and it is these parts alone that excite muscular contractions. Charles‐Édouard Brown‐Séquard (1817‐1894) experimented on the spinal cord of animals, relating epilepsy to spinal cord lesions, and used Halls’ theory and others of the time to create a new model of epileptogenesis. He observed that three or four weeks after spinal hemisection, animals showed convulsions in the nonparalyzed parts of the body including the face, reminiscent of epileptic attacks. These would begin spontaneously or by stimuli applied to the skin. This seemed to him to be a case of reflex action, and he concluded that epileptiform convulsions might be caused by slight irritation of certain nerves and suggested that blockade of the reflex arc through application of ligatures or sectioning of nerves be used as treatment.^2^^*,^ 10 ^*,^ 37 ^*^ Astly Cooper's (1768‐1841) animal experiments and Friedrich Gustav Jakob Henle (1809‐1885) determined a connection between loss of consciousness, convulsions, and change in blood supply to the brain. Another theory was that convulsions in animals were due to changes in the molecular state of the brain through malnutrition and poisoning.^2^^*^

During the age of Jackson, the definition of seizures became more closely tied to the understanding of its pathophysiology, and it seemed to finally become more widely accepted that the origin of seizures was in the brain. Richard Bright (1789‐1858) attempted to explain his clinical observations with anatomical findings, associating the symptoms of impaired sight, paresthesia, and weakness of the convulsed parts with preserved consciousness, with local lesions affecting the surface of the brain on the side opposite to the one convulsed. Todd believed that a disturbance of the hemispheric lobes may in some degree contribute to the development of convulsions. Samuel Wilks (1824‐1911) extended these ideas to say that morbid changes in the cortex of the brain accounted for almost all cases of epilepsy whether partial or generalized.^2^^*^ In 1873, Jackson defined epilepsy (or rather what we would think of today as the definition of a seizure) as the name for “occasional, sudden, excessive, rapid and local discharges of grey matter.” Based on his complicated theory on the pathophysiology of seizures, he divided the brain into three levels and characterized different seizure types based on which level the seizure was thought to originate. Discharges can start from any level and can spread to cells of the same level or different levels.^38^

Jackson's work was furthered by his colleagues and contemporaries. The discovery of the motor strip on the cortex by Gustav Fritsch (1838‐1927) and Eduard Hitzig (1838‐1907), coupled with David Ferrier's (1843‐1928) work on the anatomical description of conductive fibers, substantiated Jackson's theory about the spread of focal seizures.^9^^*,^ 10 ^*^ William Gowers (1845‐1915) focused on convulsions in which there was no visible abnormality of the brain and concluded that this type of epilepsy (idiopathic) may be explained by the discharge of gray matter, which in most cases is within the cerebral hemispheres, probably the cerebral cortex, although may be lower down such as in the medulla oblongata.^2^^*,^ 39, 40

The introduction of EEG changed the field of epileptology and was the first direct access to studying the function of the brain, and the understanding of the pathophysiology of seizures was finally becoming closer to what we “know” today. Gustav Fritsch (1838‐1927) and Eduard Hitzig (1838‐1907) induced a seizure in a dog by electric stimulation. Pavel Yurevich Kaufman (1877‐1951) and Vladimir Vladimirovich Pravdich‐Neminsky (1879‐1952) were said to be the first to associate epileptic attacks with abnormal electrical discharges on EEG.^1^^*^ Hans Berger (1873‐1941) developed the human EEG and was apparently the first to demonstrate neural oscillations in the human brain. The Gibbs’ along with Lennox redefined epilepsy (or seizures) as a “paroxysmal cerebral dysrhythmia” based on their work studying different EEG patterns.^9^^*,^ 25 In studying generalized spike and wave on invasive EEG recordings, Wilder Penfield (1891‐1976) introduced the concept of “centrencephalic” seizures produced by a circuit involving the cerebral cortex and thalamus.^41^

Cellular and molecular advances in the 20th century provided further insight into the production of seizures. This includes the use of animal models revealing that interictal discharges are associated with a paroxysmal depolarization shift and a superimposed burst of high‐frequency spikes in the cortical neurons and the evidence that generalized ictogenesis is related to hyperactivity in physiological functional anatomical networks as a result of an abnormal interaction of both cortical and subcortical mechanisms.^21^, ^42^ This new knowledge was reflected in the 2010 seizure classification revision. The concept of a network was introduced; generalized seizures originate at some point within, and rapidly engaging, bilaterally distributed networks, and focal seizures originate within networks limited to one hemisphere.^30^, ^31^ In 2012, new insights into ictogenesis led to the hypothesis of “system epilepsies,” postulating that the propensity to generate seizures of some epilepsies is due to the susceptibility of an identifiable neural system made up of brain areas, and goes beyond the simple dichotomy between focal and generalized epilepsy. This concept is differentiated from the epilepsies resulting from the sequential propagation of a discharge originating in a relatively circumscribed area to other brain areas.^43^ These relationships continue to be investigated, and it remains to be seen how these concepts may be reflected in future classifications. It is no doubt that the current definition of seizures will continue to evolve as we make advancements in our pathophysiologic understanding.

## 4 DEFINITION OF EPILEPSY

The most current definition of epilepsy has been put forth by the ILAE Definitions Task Force in 2014. Epilepsy is a *disease* of the brain defined by at least two unprovoked (or reflex) seizures occurring more than 24 hours apart, one unprovoked (or reflex) seizure with a probability of further seizures similar to the general recurrence risk (at least 60%) after two unprovoked seizures, occurring over the next 10 years, or a diagnosis of an epilepsy syndrome. It was felt that the previously used term “disorder” lacked public understanding and implied a functional disturbance, rather than a long‐lasting and serious derangement.^44^ The term “disorder” may also indicate a less severe condition that does not merit attention by health policy agencies, and if we are ever to develop a cure, the need for better communication among ourselves, our clinical colleagues, and the public is of utmost importance.

As mentioned previously, the terms used to signify seizures as a symptom and epilepsy as a disease were interchangeable in the historical literature. We will attempt to focus on the definition of epilepsy in this section, selecting from the literature the inferences that reflect our current meaning of epilepsy as a disease.

The word *epilepsy* is derived from the Greek word *epilambanein* and means “to be seized.” This was used to connote both the disease and the single attack.^2^^*^ The term signifies the magical thinking of that time that people with epilepsy were considered unclean or evil, and created the stigma related to epilepsy.

During the time of Hippocrates, epilepsy was for the first time viewed scientifically and thought to originate from the brain, rather than provoked by supernatural causes, marking the beginning of thinking about epilepsy as a medical disease. Physicians and philosophers of the post‐Hippocratic era defined epilepsy based on the clinical symptoms of the attack, and therefore, definitions varied accordingly. The definition of epilepsy as a “convulsion of the whole body together with an impairment of the leading functions” is suspected to date back to Erasistatos. This restrictive definition excluded various behaviors that were earlier considered being epileptic, namely what we call today absence and focal seizures. This definition though remained essentially unchallenged until the Renaissance.^2^^*,^ 9 ^*^

It was not until the 16th century that the existing definition of epilepsy was challenged with new clinical observations and the idea of focality emerging in the medical literature. Marcus Marci (1595‐1667) defined epilepsy as a disease where the victims are disordered in their minds and their body parts, be it all, some, or only one, move against their will.^2^^*^ Willam Cullen further refined the definition of epilepsy during the period of Enlightenment, but this continued to be based on clinical observations. He explains that epilepsy “may be defined, as consisting in convulsions of the greater part of the muscles of voluntary motion, attended with a loss of sense, and ending in a state of insensibility and seeming sleep,” but may also involve “convulsions which are…more partial: that is, affecting certain parts of the body only, and by their not being attended with a loss of sense, nor ending in such a comatose state as epilepsy always does.”^45^^*^ The work done by Jackson and his forerunners led to defining epilepsy by the pathophysiological substrate of a seizure, thus still using the symptom to define the disease.

With the advent of EEG, epilepsy was becoming recognized as a disease comprised of a specific seizure type or multiple different types of seizures. Henri Gastaut (1915‐1995) defined major human EEG patterns and focused on the recognition of specific epilepsy syndromes. His work led to the description of many important syndromes including photoparoxysmal epilepsy, startle epilepsy, hemiconvulsive‐hemiplegic epilepsy, severe encephalopathy of children with epilepsy (Lennox‐Gastaut syndrome), and benign occipital epilepsy of childhood.^1^^*,^ 9 ^*^

It seems that somewhere along the way of these new discoveries, the lines became less blurred between the distinction of seizures and epilepsy, recognizing that epilepsy is a *disease*, and seizures are the *symptom*. This is evidenced by Gastaut's early classification of both seizures and the epilepsies.^27^, ^46^ Throughout the course of multiple revisions of the classification schemes spanning over half a decade, the definition of epilepsy continues to be debated. The concept of an epileptic syndrome was introduced in the 1981 classification scheme and defined as an epileptic disorder characterized by a cluster of signs and symptoms, which may be clinical or detected by ancillary studies, typically occurring together. There is not necessarily a common etiology and prognosis as with a disease; however, some syndromes may be of great prognostic significance.^47^ In the Glossary of Descriptive Terminology for Ictal Semiology introduced in 2001 by the ILAE Task Force on Classification and Terminology, the term epilepsy is used to mean: a) an epileptic disorder defined as a chronic neurological condition characterized by recurrent epileptic seizures, or b) epilepsies defined as those conditions involving chronic recurrent epileptic seizures that can be considered epileptic disorders.^4^ In 2005, the ILAE and the IBE came to a consensus definition of epilepsy as a disorder of the brain characterized by an enduring predisposition to generate epileptic seizures and by the neurobiologic, cognitive, psychological, and social consequences of this condition. The definition requires the occurrence of at least one epileptic seizure, but does not require that the seizure be “unprovoked.”^33^ The most current definition proposed by Fisher in 2014, in addition to replacing the term “disorder” with “disease,” also recognizes the imprecise borders of provoked and unprovoked seizures, places no burden on the treating physician to specify recurrence risk in a particular circumstance, and incorporates that if there is evidence for an epilepsy syndrome, then epilepsy must be presumed to be present.^44^

For any medical entity, clear definitions are important for communication, diagnosis, and treatment. While the diagnosis of epilepsy and the decision to treat are related, but different issues, the definition we use of epilepsy may have treatment implications. The definition of epilepsy can also have socioeconomic consequences from the viewpoint of the patient, with the hope that it will lead to improved management of individuals who may have future seizures.^44^

## 5 ETIOLOGIES

Once the diagnosis of epilepsy is established, it is fundamental to identify the underlying etiology. There was a major goal of the 2017 ILAE classification of the epilepsies, where the etiological groups include structural, genetic, infectious, metabolic, immune, and unknown. The groups are not hierarchical. A patient's epilepsy can also be classified into more than one group.^48^ In going back again through our journey in time, we will see that some of these categories have been speculated since ancient times, while others emerged concurrently with expanding observations and the growing knowledge of the pathophysiology of seizures.

It seems that people living as early as in the prehistoric times may have been aware of the existence of epilepsy. Controversy surrounds the connection of prehistoric skull trepanation and epilepsy. Archeological evidence supports the theory that the practice was widespread, with skull specimens found across the world in several different continents. The earliest examples reportedly date back to perhaps the late Paleolithic period, but certainly to the Neolithic age.^49^ Whether the procedure was used for ritualistic or therapeutic purposes remains unknown. There have been speculations that it was used as a remedy for epilepsy, motivated by the then common explanation that epilepsy was caused by demons or evil spirits and that the opening of the head allowed such spirits to escape from the body.^50^

Magical thinking and supernatural beliefs dominated as the cause of epilepsy before the Hippocratic era. Aside from the moon, Greek astrological literature implicated planets such as Saturn, Mars, and Mercury, in epileptic states to create maniacs, ecstatics, and persons liable to fall. The author of *On the Sacred Disease* discussed various factors of the origin of epilepsy. “Hidden” causes, due to the lack of anatomical or physiological understanding, were differentiated from “evident” causes. Among the many “evident” causes included hereditary, sexual life, climatic factors such as changes in wind and temperature, dietetic (or regimen, a broad term in ancient medicine that covered all the necessities of daily life, including food, drink, sleep, exercise, and mental and sexual activity) irregularities, and overwhelming fright and anger in children. Asclepiades (circa 100 BC) explained epilepsy as the result of a blow and tearing the membrane covering the brain, or as the result of great fear.^2^^*^ Soranus (98‐138 AD) shared this solidary pathology and implicated meninges, contusions, and mechanical causes of epilepsy,^51^^*^ perhaps eluding to posttraumatic or structural etiologies.

The scientific setback during the Middle Ages led medieval physicians to adopt the approach of the general public and concede to the magical thinking. The rise of Christianity furthered the magical attitudes, and an important example of this can be seen in a story in the New Testament (Mark, 9:14‐29; Matthew, 17:14‐20; Luke, 9:37‐43), which describes a boy with epilepsy (termed a “lunatic”) who Jesus cures by driving out of him the “foul spirit.” The connection between epilepsy and supernatural powers is also exemplified in the association with certain saints, notably St. John (*le mal Saint‐Jean* was a common expression in France), St. Valentine (whose name and the German word *fallen* sounded identical), and St. Vitus (St. Vitus’ dance, which interestingly is now mostly associated with chorea). Dante's *Inferno* describes a seizure as due to demons. The infectious nature of epilepsy and the fear of catching the disease were clearly voiced during this time.^2^^*,^ 11 ^*^ Ali Ibn Abbās (lived during the Islamic Golden age, died 982/994 AD), a Persian physician, proposed that skull fractures caused compression of the brain resulting in epilepsy,^2^^*^furthering the idea of a posttraumatic or structural etiology.

The debate around the causes of epilepsy continued through the Enlightenment period, with seemingly few new ideas. Herman Boerhaave (1668‐1738) stated the causes of epilepsy are hereditary or from the imagination of the mother when she is pregnant being shocked at the sight of a person in an epileptic fit. It occurred in abnormally shaped skulls where foul fluid was stagnant, which caused an excess of water accumulation in the brain.^2^^*^ Tissot refuted the influence of the moon and pregnancy, believing that the brain is solely responsible for epilepsy. He brought back the notion that sexual excess or masturbation could be a cause of epilepsy.^52^^*^ A distinction was made between the predisposing and provoking causes of epilepsy, with relatively little discussion on the former. Provoking factors included “passions,” shock, overwork or fright, tumors, skull fractures, brain hemorrhages, hardening of the cerebral hemispheres, syphilis, and fever among various others of which were greatly aided by descriptions of abnormalities in the field of anatomy.^2^^*,^ 11 ^*^

The structural cause of epilepsy was highlighted during the age of Hughlings Jackson. In describing cases of syphilitic epilepsy, his observations centered around unilateral convulsions, and his anatomical investigations showed the cause was obvious organic disease on the side of the brain opposite to the side of the body convulsed,^53^ a conclusion set forth by some of his forerunners. Jackson's collaboration with surgeon Victor Horsley (1857‐1916) has been said to have marked the birth of epilepsy surgery and the closely related new term “focal.” A patient of theirs was operated upon based on the anatomical conclusion from seizure semiology; Horsley removed a tuberculoma from a region of the cortex, which he and Jackson considered the “epileptogenic focus,” and the patient became seizure‐free.^54^ The use of EEG became important for localization for surgical treatment of intractable epilepsy. Penfield and Herbert Jasper (1906‐1999) introduced EEG as a routine method in neurosurgery. The rise of neuroimaging over half a century later further supported Jackson's structural theory of epilepsy. The invention of computerized tomography (CT) in 1972 by Godfrey Hounsfield (1919‐2004) and Allan MacLeod Cormack (1924‐1998) allowed for gross lesions to become visible for the first time. Magnetic resonance imaging (MRI) was introduced in the 1980s and made a greater impact, allowing for the identification of even subtle brain lesions. Cases once considered “cryptogenic” (of suspected, but not identified cause) were being classified as symptomatic. Further techniques including functional MRI, positron emission tomography (PET), single‐photon emission computed tomography (SPECT), and magnetoencephalography (MEG) continued to make significant contributions to the detection of epileptogenic lesions. Along with this came the rise of epilepsy surgery and the use of modern techniques such as intracranial EEG and stereoelectroencephalography (sEEG) to precisely localize the epileptogenic zone.^9^, ^42^ Neurosurgical methods have now advanced far beyond resection, employing to various extent procedures such as callosotomy, multiple subpial transection (MST), vagal nerve stimulator (VNS), or more recently responsive neurostimulator (RNS) for nonresectable epileptogenic lesions. The trend in all forms of surgery toward minimally invasive techniques has led to the exciting modern development of neuroablation to treat focal lesions in deep parts of the brain.^42^, ^55^

The genetics of epilepsy seemingly first came to light in 1903 by Herman Bernhard Lundborg (1868‐1943) who published his research on the genetics of progressive myoclonic epilepsy first described by Heinrich Unverricht (1853‐1912), by tracing the disease to one extensive kindred back to the 1700s. An explosion in genetic techniques over the last several decades identified the specific genetic mutation associated with the disease and has also implicated many other genetic mutations in a number of human epilepsy syndromes.^9^^*^ A hereditary cause for epilepsy has been speculated since the time of Hippocrates; however, “genetic” does not necessarily mean “hereditary,” and only with these modern advancements do we better understand this as a cause for epilepsy.

Autoimmune‐mediated epilepsy as an etiology only became notable in the second half of the 20th century. In the 1960s, Brierley, Corsellis, and colleagues described patients with a subacute onset of amnesia, disorientation, and seizures with histological evidence of limbic system inflammation.^56^, ^57^ The association with systemic malignancies later became understood as more cases were observed. There has been a recent upsurge of this field over the past several years as new autoantibodies are being discovered.^58^

As we now circle back to modern day, we realize how important it is to identify the etiology of one's epilepsy, as this is what will ultimately guide treatment and determine prognosis. While some of the etiologies we identify today have been suspected since ancient times, others have only become apparent with more recent scientific and technological advances.

## 6 CLASSIFICATION SCHEMES

The classification of epilepsy is intimately tied to seizure semiology, pathophysiology, and etiology, which becomes evident as we see how classification schemes have evolved over time. It is important to note again that the distinction between seizures and epilepsy was not clear in early times. The classification of seizures was discussed above, and so this section will only briefly touch upon this to understand the trajectory over time, but focus on the classification of the epilepsies.

The current epilepsy classification was proposed in 2017. A multilevel classification was designed. The starting point is the seizure type as outlined in the accompanying proposal by Fisher et al, 2017. This is followed by the epilepsy type, which includes focal, generalized, unknown, and a new category for combined generalized and focal epilepsy. The third level of classification is an epilepsy syndrome diagnosis. Etiological classification, endorsed in the 2010 classification, was expanded and included genetic, infectious, metabolic, immune, and unknown groups. Lastly, the importance of the presence of comorbidities such as learning, psychological, and behavioral problems was acknowledged, and should be considered for every patient with epilepsy at each stage of the classification.^48^

The earliest reported classification schemes were based predominantly on clinical manifestation. Galen first dichotomized seizures as those originating from the brain (“idiopathic” or “protopathic”) versus other body parts with subsequent involvement of the brain (“sympathetic”). This was subdivided into originating from the cardia (relating to the stomach) or any other part of the body. Despite the scientific setback during the Middle Ages, Galenic views endured in the medical writing though some new terms were introduced. Epilepsy was the term designated to the idiopathic form of the disease, “analepsy,” referred to seizures arising from the stomach, and “catalepsy” was used to refer to seizures arising from another part of the body. Platearius (12th century), a Salernitan physician, additionally distinguishes between “major” and “minor” epilepsy. He described “major epilepsy” as a complete obstruction of the principal ventricles of the brain, clinically characterizing this as a full convulsion, and “minor epilepsy” as an incomplete obstruction of the ventricles of the brain characterized by milder symptoms, though the distinction between the two was not always so clearly defined. John of Gaddesden (14th century), an English physician, established three forms of epilepsy: minor (or true, resulting from obstruction of the arteries), medium (or truer, resulting from obstruction of the nerves), and major (or truest, resulting from obstruction of the ventricles of the brain).^2^^*^

During the Renaissance, the concept of “symptomatic” epilepsy came to light, where epilepsy was a manifestation or complication of another disease, not actually a disease itself. During Enlightenment, Galen's division of idiopathic and sympathetic epilepsy prevailed. However, it was questioned as to how to classify those cases where no definite cause was discovered. Tissot called these cases “essential” epilepsy. Idiopathic epilepsy was named “cerebral epilepsy,” while the many forms of sympathetic epilepsy were named by their origin (“stomachica,” “splenetica,” “nephritica,” “hysterica,” etc). Boissier de Sauvages (1706‐1762) collected all such forms and classified them as subdivisions. Cullen subdivided idiopathic epilepsy into: (1) *Epilepsia cerebralis* as suddenly coming on without manifest cause, (2) *Epilepsia sympathica* as also without manifest cause but preceded by an aura, and (3) *Epilepsia occasionalis* as arising from manifest irritation, with there being many diverse irritations and containing a multitude of causes of epilepsies (ie, from head injury, poison, affection of the mind).^2^^*^

In the 19th century, there were further attempts at nosological classification, although with the diverse theories on the pathophysiology and etiology of epilepsy, these ideas still seemed to be scattered. Louis Maisonneuve (1745‐1826) assigned five species to each of the two traditional subdivisions; idiopathic epilepsy was congenital, spontaneous, plethoric, and humoral, or caused by strong emotions, whereas sympathetic epilepsy was produced by “irradiation” from external parts, the stomach, intestines, or uterus, to which “vaporous or hypochondric epilepsy” was added as well.^2^^*^ Sympathetic epilepsy was doubted by a few, including Etienne‐Jean Georget (1795‐1828) who stated he had never observed such a case of sympathetic epilepsy and supported his older predecessors that all epileptic attacks originated in the brain.^10^^*^ Louis Delasiauve (1804‐93) proposed the following: 1. *Essential or idiopathic epilepsy*, manifesting as functional deviations, without a lesion and corresponding to simple nervous afflictions; 2. *Symptomatic epilepsy*, due to more or less an appreciable cerebral lesion, with the seizure being the symptom and not the disease; and 3. *Sympathetic epilepsy*, produced by irradiation of abnormal impressions, which can have their seat in all parts of the body except the brain or its appendages.^2^^*^

With the breakthroughs made during the age of Jackson, classification schemes seemed to have introduced a dichotomy between focal and generalized, and also elaborated on etiology, though still far from our current classifications today. At the end of the 19th century, Charles Féré (1852‐1907) was the first work to discuss “epilepsies” in the plural form. He distinguished between partial and generalized paroxysms; the latter were subdivided into complete, incomplete, abnormal, or isolated.^21^^*,^ 59 ^*^ On the other hand, William Aldren Turner (1864‐1945) focused on idiopathic epilepsy; however, he did not clearly define what he meant by “idiopathic,” other than excluding patients with any recognized organic disease of the brain, thereby disregarding Jacksonian epilepsy. He also extended the range of psychical manifestation of epilepsy and was an advocate of psychical “epileptic equivalents.” In his final remarks on the topic of epilepsy, he classified the disease on the basis of etiology into organic, toxic/infective, psychogenic, and unknown origin.^40^^*,^ 60, 61 ^*^

The development of the EEG aided in the first attempt of the international classification of epilepsy led by Gastaut in 1969, following his classification of seizures. He distinguished three major groups: Generalized epilepsies further subdivided into primary generalized (generally corresponding to the common, essential, genuine, idiopathic, or true epilepsy of older authors, and to the centrencephalic epilepsy of modern authors; and absence of neurological or psychiatric evidence of cerebral involvement interictally) and secondary generalized (of which seizures were generalized from the start or initial focal onset not apparent, and with evidence of neurological, psychiatric, and radiological signs of diffuse cerebral involvement such as West syndrome or Lennox syndrome). The second group was partial (or focal) epilepsies characterized by seizures whose symptoms take on very different forms according to the functions of the neuronal population where the discharge originates, and which relatively frequently had the presence of an epileptogenic lesion. The last group was unclassifiable. Within each group, there were several criteria including clinical and EEG manifestations of the seizures, interictal EEG, age at onset, presence or absence of interictal neuropsychiatric changes, response to therapy, presence or absence of a more or less evident etiology, and a known or supposed pathophysiology.^46^ In 1970, JK Merlis adopted and modified this classification, with hopes of use by all physicians and not just epileptologists.^62^ Objections were raised against this classification system as it was thought unlikely that the general physician would find it practicable. Given the present state of knowledge of epilepsy, some felt that an all‐embracing classification was impossible.^63^

Following a revised classification of seizures in 1981, there was a revised classification for epilepsies and epilepsy syndromes, the aim of which was to provide a scheme that would be compatible with the view of the majority of epileptologists and allowing for mutual exchange of ideas. A dual dichotomy scheme was used; the first divided epilepsy by semiology into generalized or localization‐related partial or focal, and the second divided epilepsy by etiology into symptomatic or “secondary” and idiopathic (primary) or cryptogenic. Idiopathic epilepsies were grouped according to age of onset. A third category for undetermined epilepsies was also added.^64^ The ILAE revised their proposal in 1989 with the key feature being the addition of a group of cryptogenic epilepsies. This term was used to refer to a disorder whose cause is hidden or occult, to avoid symptomatic epilepsies being classified as idiopathic when the cause could not be identified.^65^

With the growing understanding of seizures and epilepsy brought about by the scientific and technological advances beginning in the second half of the 20th century came the need for further revisions and proposals of classification schemes. The ILAE Task Force on Classification and Terminology in 2001 proposed a diagnostic scheme within which a variety of approaches to classification are possible. As mentioned earlier, they suggested a five‐axis scheme: 1) ictal semiology using a standardized Glossary of Descriptive Terminology; 2) seizure type or types, derived from a list of accepted seizure types, with localization specified when this is appropriate, and in the case of reflex seizures, the specific stimulus; 3) syndrome derived from a list of accepted epilepsy syndromes, understanding that this may not always be possible; 4) etiology when this is known derived from a classification of diseases frequently associated with epileptic seizures or syndromes; and 5) optional designation of degree of impairment caused by the epileptic condition, derived from the World Health Organization ICIDH‐2 International Classification of Functioning and Disability. This work though did not negate the 1989 classification of epilepsies. Therefore, it was agreed that these classifications would not be discarded unless, or until, a clearly better classification was contrived. An update was issued in 2006, which was basically unchanged from the previous classifications, except the list of epileptic syndromes was revised.^66^

The next major revision was issued in 2010. A primary motivation was to have the classification of seizures and epilepsies reflect all of the advances made in basic and clinical neurosciences, so that this could be incorporated into clinical practices. The terms idiopathic, symptomatic, and cryptogenic were to be replaced with more specific terms from an etiological standpoint including genetic, structural/metabolic, or unknown. These categories were not mutually exclusive. The concept of an electroclinical syndrome was re‐established; the term “syndrome” was to be restricted to a group of clinical entities reliably identified by a cluster of electroclinical characteristics, and the term constellation was to be used for entities, which are not exactly syndromes but are diagnostically meaningful forms of epilepsy and may have implications for clinical treatment such as mesial temporal lobe epilepsy. The group concluded that no one specific organization was proposed for the revised classification, but rather, the various forms of epilepsy were to be organized according to those aspects that were most pertinent to a specific purpose.^30^ The changes made in the classification of seizures and epilepsies invoked debate and controversy. This included the continuous lack of complete scientific understanding in epileptogenesis to support such a classification scheme, presumed failure to incorporate known advances in the field, and criticism of the new terminology that some thought to be unnecessary. Others found that it was too difficult to incorporate into daily clinical practice.^21^, ^31^, ^32^ This ultimately led to the current classification scheme described earlier in this section.

For this most current classification, the ILAE introduced a new methodology approach; they used the Internet to solicit public comments and criticism and appointed a separate expert panel to review these remarks, rather than ratification by the General Assembly through a vote by the representatives of the ILAE Chapters from around the world. While no classification scheme will be perfect at this point, the current proposed scheme was fairly well received. It took a necessary step toward aligning clinical practice with scientific advances in the field of epileptology. Currently, ongoing trials of pharmacological and surgical therapies highlight the importance of etiology‐driven classification. Some remarks included that the classification was limited by the attempt to box cases as seen in clinical practice into categories with no room for variants or atypical clinical presentations, the lack of boundaries of epilepsy syndromes or to what extent syndromic variants should be included, and ill‐defined comorbidities.^67^, ^68^, ^69^ Other recent critiques included the mixture of semiological terms with epileptogenic zone terminology, replacement of simple and widely accepted terminology with complex terminology containing less information, the limitation of describing seizure evolution in any detail, and the 100% overlap between seizure type and epilepsy type.^70^ The ILAE responded that until science explains why there are different types of seizures, every classification will be a compromise reflecting consensus and pragmatism. The task force continues to believe that the new classification scheme is a significant step forward in the large majority of patients with epilepsy.^71^

## 7 CONCLUSION

This review aims to objectively summarize the chronological development of the concepts of seizures and epilepsy, from the earliest notion of the disease to the most current views. While it is not feasible to discuss every historical figure or detail every classification proposal, we hope the reader has been transported through this evolutionary journey to understand the major milestones in the field. It is evident that those living as far back as 2500 BC were aware of seizures and provided descriptions in line with how we would describe them today. Through the years, these descriptions have been revised, updated, and recycled, and new terminology has been assigned, in a manner that some would say is just a matter of semantics. However, what is remarkable and clearly manifest is how far we have come in understanding the pathophysiology and etiology of the disease, from magical beliefs, to the idea of the origination in the brain and excess humors, to our current perceptions today.

Classification will continue to be a dynamic process, reflecting new knowledge gained through research and scientific advances. It will undoubtedly engender debate, until our understanding is advanced enough to create a classification scheme based predominantly on scientific grounds. It is hoped that it is relevant to clinical practice and serves as a common language. Importantly, these classification schemes should lead to improved diagnosis and understanding of etiology, and ultimately guide targeted treatments.

## CONFLICTS OF INTEREST

Dr Moshé is serving as Associate Editor of Neurobiology of Disease and is on the editorial board of Brain and Development, Pediatric Neurology and Physiological Research. He receives from Elsevier an annual compensation for his work as Associate Editor in Neurobiology of Disease and royalties from 2 books he coedited. He has received consultant's fees from UCB, Mallinckrodt, and Pfizer. Dr Patel has no conflicts of interest. We confirm that we have read the Journal's position on issues involved in ethical publication and affirm that this report is consistent with those guidelines.
